# Role of S1PR1 in Modulating Airway Epithelial Responses to *Pseudomonas aeruginosa* in Cystic Fibrosis

**DOI:** 10.3390/pathogens14111146

**Published:** 2025-11-12

**Authors:** Cristina Cigana, Claudia Caslini, Alessandro Migliara, Beatriz Alcala’-Franco, Laura Veschetti, Nicola Ivan Lorè, Angelo Lombardo, Alessandra Bragonzi

**Affiliations:** 1 Infections and Cystic Fibrosis Unit, Division of Immunology, Transplantation and Infectious Diseases, IRCCS San Raffaele Scientific Institute, 20132 Milan, Italy; cigana.cristina@hsr.it (C.C.); caslini.claudia@hsr.it (C.C.); alcalafranco.beatriz@hsr.it (B.A.-F.); veschetti.laura@hsr.it (L.V.); lore.nicolaivan@hsr.it (N.I.L.); 2 San Raffaele Telethon Institute for Gene Therapy, IRCCS San Raffaele Scientific Institute, 20132 Milan, Italy; migliara.alessandro@hsr.it (A.M.); lombardo.angelo@hsr.it (A.L.); 3 Vita-Salute San Raffaele University, 20132 Milan, Italy

**Keywords:** sphingosine 1-phosphate receptor 1, cystic fibrosis, modifier genes, *Pseudomonas aeruginosa*

## Abstract

**Background:** *Pseudomonas aeruginosa* infection is a major driver of morbidity and mortality in cystic fibrosis (CF), yet disease severity varies widely among people with CF (pwCF). This clinical heterogeneity suggests the involvement of host genetic modifiers beyond *CFTR*. We previously identified *sphingosine 1-phosphate receptor 1* (*S1PR1*) as a candidate gene associated with susceptibility to *P. aeruginosa*. Here, we investigated its role in modulating airway epithelial responses to infection. **Methods:** Using CRISPR/Cas9, we generated S1PR1-knockout bronchial epithelial cells with (IB3-1) and without (C38) *CFTR* mutations. We assessed cell viability, cytotoxicity, and interleukin-8 secretion following exposure to *P. aeruginosa* exoproducts. S1PR1 protein expression was evaluated in lung tissue from pwCF and non-CF individuals using immunohistochemistry. **Results:** S1PR1-mutant cells produced truncated, non-functional peptides. In CFTR-mutant cells, S1PR1 loss reduced viability, increased cytotoxicity, and significantly enhanced interleukin-8 production in response to *P. aeruginosa* exoproducts. These effects were not observed in CFTR-competent cells. Notably, S1PR1 protein levels were markedly lower in lung tissue from pwCF compared to non-CF individuals. **Conclusions:** S1PR1 deficiency exacerbates epithelial damage and inflammatory responses to *P. aeruginosa* in CF models. These findings highlight S1PR1 as a potential contributor to infection severity and a promising target for therapeutic strategies in pwCF.

## 1. Introduction

The identification of modifier genes contributing to individual risk factors for lung pathology is both a clinical necessity and a forthcoming challenge in achieving effective treatment for cystic fibrosis (CF). CF presents particular challenges due to the complex relationship between genotype (CFTR function) and phenotype (clinical outcome) [1]. The disease phenotype primarily manifests as lung and exocrine pancreatic insufficiency, liver fibrosis, and male infertility. The absence of functional CFTR protein in the airways leads to airway surface dehydration, impaired mucus clearance, increased susceptibility to infections, and inflammation [2]. Since the early descriptions of CF, *Pseudomonas aeruginosa* has been recognized as the predominant respiratory pathogen in this disease, with pulmonary exacerbations remaining a major cause of morbidity and mortality [3]. With the advent of new modulator therapies, the landscape of CF has evolved significantly, improving clinical outcomes and extending life expectancy, yet *P. aeruginosa* continues to be a significant respiratory pathogen [4].

The clinical outcome of *P. aeruginosa* airway infections varies significantly among people with CF (pwCF) with the same *CFTR* mutation, ranging from mild to severe life-threatening conditions [5]. This clearly indicates that the CFTR genotype alone cannot account for the diversity of the lung phenotype [6]. Before extensive genetic data became available, the prevailing concept attributed the heterogeneity of the clinical lung phenotype primarily to environmental influences, including the risk of acquiring different types of pathogens with varying pathogenic potential. However, the role of host genetics was somewhat underestimated as a contributing factor to this complexity. In recent years, thanks to human genome sequencing, there has been a growing interest in understanding genetic variations, commonly referred to as “modifier genes,” which can influence the CF phenotype [5,7].

To complement human genetic studies, we employed animal models to identify potential disease modifiers. We previously demonstrated that the genetically diverse Collaborative Cross mouse model population exhibits a wide range of heritable responses to respiratory *P. aeruginosa* infections [8,9]. This mouse population serves as a valuable tool for identifying genetic disease modifiers, encompassing multiple genetic loci, or polymorphic variations that affect morbidity and mortality in *P. aeruginosa* pneumonia. Based on significant changes in survival times, a quantitative trait locus (QTL) consisting of 31 protein-coding genes was mapped. Within this locus, sphingosine-1-phosphate receptor 1 (*S1PR1*) was identified based on genome-wide significance and disease gene prioritization. *S1PR1* encodes a G-protein-coupled receptor (S1PR1) that binds the bioactive signaling molecule sphingosine-1-phosphate (S1P) and plays a role in various physiological processes, including inflammation mediated by viral and bacterial infections [10,11]. S1PR1 is expressed in a variety of cell types, such as endothelial and epithelial cells, T cells, natural killer T cells, innate lymphoid cells, and dendritic cells [12,13,14]. In humans, 15 nonsynonymous *S1PR1* SNPs have been identified by the U.S. National Heart, Lung, and Blood Institute Exome Sequencing Project [15]. Although specific pathogenic mutations in the *S1PR1* gene have not been reported in pwCF, several lines of evidence point to its potential role as a modifier gene influencing the pulmonary phenotype. In particular, functional S1PR1 variants have been implicated in chronic respiratory disorders, including asthma [16], idiopathic pulmonary fibrosis [17], and acute lung injury [18], supporting the idea that genetic variants of *S1PR1* may contribute to disease heterogeneity and inflammatory dysregulation in the lungs. Functional validation of the S1PR1 through pharmacological targeting in C57BL/6NCrl mice confirmed its relevance in the pathophysiology of *P. aeruginosa* infections [9]. Furthermore, the bioactive signaling molecule S1P inhibits CFTR activity via adenosine monophosphate-activated kinase (AMPK) [19], establishing a link to CF.

Since there appears to be a relationship between S1PR1 and CFTR, we explored the specific impact of *S1PR1* mutations on epithelial cytotoxicity and inflammation within the CF context. Through the creation of a novel cellular model, we demonstrated that *S1PR1* mutation, in combination with CFTR dysfunction, enhances cytotoxicity both under basal conditions and upon stimulation with *P. aeruginosa* exoproducts. This alteration also amplifies the inflammatory response triggered by *P. aeruginosa* exoproducts. Furthermore, our investigation of lung tissues from pwCF revealed a conspicuous absence or significant reduction in S1PR1 protein signals compared to non-CF individuals. These findings underscore the potential role of S1PR1 as a potential modifier in CF and emphasize the need for further exploration into its implications.

## 2. Materials and Methods

### 2.1. Ethics Statement

Research on the bacterial isolate from the individual with CF has been approved by the responsible physician at the CF center at Hannover Medical School, Germany. The person gave informed consent before the sample collection. Approval for the storing of biological materials was obtained by the Hannover Medical School, Germany. The use of human bronchi, obtained from pwCF who underwent lung transplant, was approved by the Ethical Committee of the Gaslini Institute (Genova, Italy), in accordance with the guidelines of the Italian Ministry of Health. Each person provided written informed consent to the study using a form that was also approved by the Ethical Committee (approval #13).

### 2.2. Bacterial Strain and pwCF

*P. aeruginosa* clinical isolate AA2 was obtained from sputa or throat swabs from a person homozygous for F508del *CFTR* mutation attending the Medizinische Hochschule of Hannover, Germany, at the onset of chronic colonization. Genotypic and phenotypic data of *P. aeruginosa* strains were published previously [20]. *P. aeruginosa* was cultured in trypticase soy broth (TSB) at 37 °C.

### 2.3. Generation and Sequence of S1PR1 Mutant Cells by CRISPR/Cas9

IB3-1 cells derived from the bronchial epithelium of a person with CF (*F508del/W1282X* genotype), and their CFTR-corrected counterpart, C38 cells, obtained by transfection with *wt CFTR* [21], were used. Cells were co-transduced with two lentiviral vectors encoding single guide RNAs (sgRNAs) at a high multiplicity of infection (MOI) in the presence of polybrene to enhance transduction. This vector also carries puromycin resistance for clone selection (Cas9.FLAG.P2A.PuroR transgene; Addgene Plasmid #52963) [22]. Subsequently, transduced cells were selected by the administration of puromycin in 6-well culture plates. Subsequently, single transduced cells were sorted using flow cytometry into separate wells of a 96-well plate. Genomic DNA from single clone cells was extracted using a DNeasy Blood & Tissue Kit (Qiagen, Milano, Italy). Deletions in the S1PR1 gene were confirmed through amplicon sequencing. Gene amplicons were obtained through PCR (Supplementary Table S1). Sequencing libraries were prepared following the TruSeq DNA Nano protocol according to the manufacturer instructions, and sequencing was carried out on an Illumina Novaseq 6000 platform (2 × 100 nt). The quality of the raw reads was assessed using FastQC v0.12.1, and adapter and quality trimming was performed using Trimmomatic v0.39 (Illuminaclip:adapter_file.fa:2:30:20 leading:3 trailing:3 slidingwindow:4:20 minlen:50). Reads were then aligned to the *Homo sapiens* S1PR1 gene sequence (RefSeq accession: NG_016181.1) using Minimap2 v2.28-r1209 using the -x splice preset. Peptide generation predictions were carried out using the Translate tool available on the Expasy platform (link: https://web.expasy.org/translate/, accessed on 5 April 2024).

### 2.4. Bacterial Exoproducts Preparation

Three overnight (O/N) liquid growths of the *P. aeruginosa* AA2 strain (deriving from individual colonies grown on tryptic soy agar plates) were refreshed in tryptic soy broth (TSB) at an optical density at 600 nm (OD_600_) of 0.1 and grown at 37 °C 200 rpm. After 4 h, the bacterial growth media were collected and centrifuged, and the pellets were resuspended in TSB, the OD_600_ measured, and the bacterial growths were refreshed at 0.1 OD_600_ in TSB and grown at 37 °C at 200 rpm. After 6 h, the bacterial growth media were merged and centrifuged. The supernatant (SN) was collected, filtered with 0.22 µm filters, aliquoted, and stored at −80 °C.

### 2.5. Cell Stimulation

IB3-1 and C38 cells (either *SP1R1*-mutated or not), grown as previously described [23], were plated at a density of 4 × 10^4^ cells/cm^2^ in duplicate in wells of 6-well plates (for the ELISA) or in quadruplicate in 96-well plates (for the Alamar assay). The day after, AA2 extracellular supernatant was used to stimulate the cells (at 10% in the cell medium, a concentration selected based on previous dose–response analyses performed in our laboratory, in 1 mL volume in the 6-well plates and 300 µL in the 96-well plates). As a control, filtered TSB was used at the same percentage. In the 96-well plates, the Alamar Blue was added to each well at a final concentration of 2%.

### 2.6. Cytotoxicity and ELISA

After 24 h from the stimulation, the cell medium was centrifuged and the SN used to quantify interleukin-8 (IL-8) release by ELISA assay following manufacturer indications (R&D Systems Duoset ELISA Development System). For the cytotoxicity assay [24], at specific time-points (20 min, and 2, 4, 6, and 24 h), 50 µL of medium were transferred to the wells of black 96-well plates, and fluorescence was read at the Victor X5 microplate reader (Perkin Elmer) (emission: 595/560). The fluorescence values obtained, subtracted of the blank, were normalized on those of naïve cells at 20 min and expressed as percentage.

### 2.7. Immunostaining Against S1PR1

Localization of S1PR1 was conducted via indirect immunofluorescence. After fixation with 4% paraformaldehyde (PFA), cells were incubated with an anti-human S1PR1 monoclonal antibody (EDG-1/S1P1/S1PR1 (A-6)|SCBT—Santa Cruz Biotechnology, Heidelberg, Germany) used at 1:200 in PBS with bovine serum albumin (BSA) 4%. Secondary antibody (1:500) was a Texas Red-labeled goat anti-mouse. Daco Fluorescent Mounting Medium was used to cover the slides. Immunofluorescence images were recorded with an EM-CCD Hamamatsu C9100 camera (Hamamatsu Photonics, Hamamatsu City, Japan) mounted on an UltraVIEW Spinning Disk Confocal Microscope (Perkin Elmer, Waltham, MA, USA). Sections of human lungs were deparaffinized and stained with the anti-human S1PR1 monoclonal antibody (1:100). Secondary antibody (1:50) was a biotinylated rabbit anti-mouse. Pierce™ Substrate Kit (Thermo Fisher, Monza, Italy) was used for revelation, followed by hematoxylin and eosin staining. Slides were visualized with Axioplan2 (Zeiss, Jena, Germany) with AxioCam provided with the CCD MRc5 (Zeiss).

### 2.8. Statistics

Statistical analyses were performed with GraphPad Prism version 9 (GraphPad Software, Inc., San Diego, CA, USA), using a one-way ANOVA test with the Šidák correction for multiple comparisons to compare IL-8 levels and a two-way ANOVA test with the Bonferroni correction for multiple comparisons to compare cell viability.

## 3. Results

### 3.1. Generation of Mutant Cell Lines for S1PR1 Through CRISPR/Cas9 Genetic Editing

Our goal was to generate a genetically defined human cell line that would enable us to investigate the role of *S1PR1* in the presence or absence of a *CFTR* mutation. Human bronchial immortalized cell lines IB3-1, which is deficient for *CFTR* (*F508del/W1282X*) and C38, which has a corrected CFTR, were engineered to introduce a mutation in the *S1PR1* gene using the CRISPR/Cas9 technique. Three *S1PR1* sgRNAs (Table 1) were designed using a bioinformatics tool that predicts the most specific and efficient guides (link: http://chopchop.cbu.uib.no/, accessed on 19 November 2017). These three sgRNAs target the only annotated coding exon of S1PR1 at different positions Specifically, W3 sgRNA targets the initial portion of the *S1PR1* gene, W6 targets the central portion, and W9 targets the final portion.

**Figure 1:**
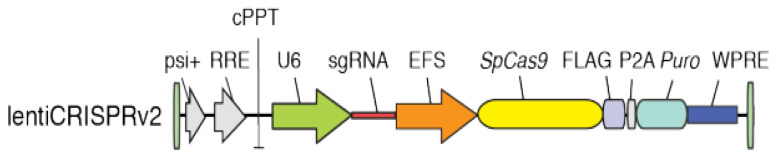
Schematic representation of the lentiviral vector encoding the Cas9 nuclease, sgRNA, and puromycin resistance, used for the transduction of the human bronchial immortalized cell lines C38 and IB3-1.

Subsequently, the three sgRNAs were cloned into a lentiviral vector that encodes both the guides and Cas9, along with the gene for puromycin resistance for clone selection [22] (Figure 1). The lentiviral vector was chosen to enable the co-expression of Cas9 and the guide of interest.

The IB3-1 and C38 cell lines were co-transduced with two lentiviral vectors encoding sgRNAs W3 and W9, while guide W6 was kept as a reserve. Subsequently, transduced cells were selected by the administration of puromycin. Using flow cytometry, individual cells were isolated, and clones were selected and grown individually for expansion to create homogeneous cell populations of IB3-1 *S1PR1^mut/mut^* and C38 *S1PR1^mut/mut^* lines. Following the same procedure, control cells were obtained, including IB3-1 *S1PR1^wt/wt^* and C38 *S1PR1^wt/wt^* cells. These cells were transduced with an empty control viral vector, lacking sgRNA. As a result, these cells express S1PR1, but since they were treated under the same conditions as the mutant clones, they serve as the best positive control.

To sequence the alleles of candidate clones, we initially amplified the *S1PR1* target locus through PCR and subsequently performed next generation sequencing. The sequencing revealed that our clones displayed various deletions. Specifically, for the C38 *S1PR1^mut/mut^* cell line, we selected Clone 23, which exhibited a 550 bp deletion after codon 13 in one allele and a 480 bp deletion after codon 12 in the other allele (Supplementary Alignments S1A and B). In the IB3-1 *S1PR1^mut/mut^* cell line, clone 8 displayed a 550 bp deletion after codon 13 in one S1PR1 gene allele and a 17 bp deletion, followed by another 455 bp deletion, after codon 13 in the other allele (Supplementary Alignments S1C and D). Bioinformatic analysis predicting peptide generation found that clone 23 (C38 *S1PR1^mut/mut^*) is expected to generate 6 and 5 peptides from each allelic sequence, whereas clone 8 (IB3-1 *S1PR1^mut/mut^*) could generate 5 and 11 peptides (Supplementary Alignments S1). When we investigated the presence of transmembrane domains in the generated peptides to assess possible functionality, we found substantial or complete deletion of these domains. The presence of frameshift mutations, premature stop codons, and the absence of functional domains in our clones determine the generation of small peptides that do not lead to functional receptors.

### 3.2. Immunolocalization of S1PR1 and Its Mutant Form in the Presence or Absence of CFTR

**Figure 2:**
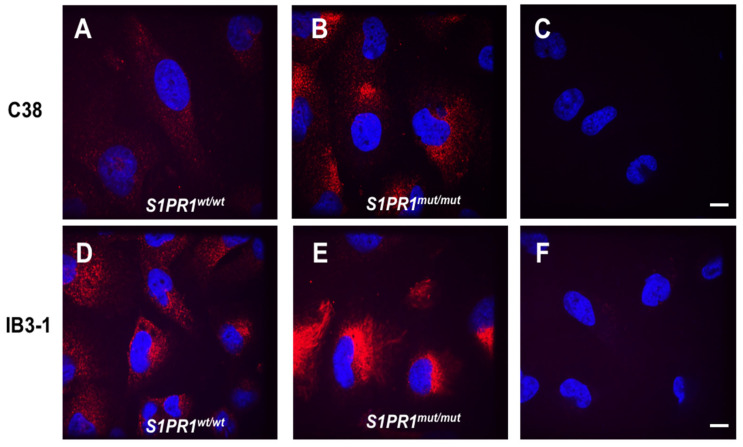
Localization of S1PR1 and its mutant form in C38 and IB3-1 cell lines. Representative images display immunofluorescence staining with anti-S1PR1 antibody (in red) in C38 *S1PR1^wt/wt^* (**A**), C38 *S1PR1^mut/mut^* (**B**), IB3-1 *S1PR1^wt/wt^* (**D**), and IB3-1 *S1PR1^mut/mut^* (**E**). The nucleus was stained with 4,6-Diamidino-2-phenylindole dihydrochloride (DAPI) (in blue) (**A**–**F**). Different localizations were analyzed using confocal microscopy (Perkin Elmer, Waltham, MA, USA). Negative control cells were stained by omitting the primary S1PR1 antibody (**C**,**F**). Scale bar: 10 µm.

The engineered cell lines generated in this work serve as models for assessing the role of the S1PR1 protein in the pathophysiology. Clone 8 (IB3-1 *S1PR1^mut/mut^*) and clone 23 (C38 *S1PR1^mut/mut^*) were chosen as representatives of all isolated clones. The immunolocalization of S1PR1 in our cell lines reveals different patterns (Figure 2). In C38 *S1PR1^wt/wt^* cells, confocal microscopy shows that S1PR1 appears to be uniformly expressed throughout the cell (Figure 2A). The mutation in S1PR1 of C38 *S1PR1^mut/mut^* cells results in a shortened protein (159 aa deletion) and other small peptides being generated by each clone allele that are still recognized by S1PR1 antibody (Supplementary Alignments S1A and B). These peptides are primarily localized to the perinuclear cytoplasmic region (Figure 2B) since they are not correctly processed, as their localization significantly differs between *wt/wt* and *S1PR1* mutant cells.

When analyzing cell lines with mutations in CFTR, we observed that in IB3-1 *S1PR1^wt/wt^* cells, S1PR1 is diffuse throughout the cell and also accumulates in a perinuclear location (Figure 2D). The mutation of *S1PR1* in IB3-1 generated several peptides of different lengths that mainly accumulate the protein predominantly in the perinuclear cytoplasmic region (Figure 2E). These findings imply that mutations in S1PR1 disrupt protein processing and trafficking, further contributing to cellular stress and pathology in addition to the effects of CFTR mutations.

### 3.3. Response of IB3-1 and C38 Cell Lines Carrying S1PR1 or Its Mutant to Stimulation with P. aeruginosa Exoproducts

**Figure 3:**
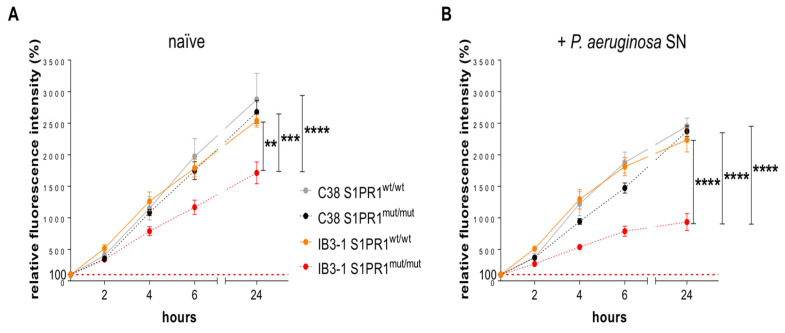
Cell viability in IB3-1 and C38 cell lines, both *S1PR1^mut/mut^* and *S1PR1^wt/wt^*. A time-course analysis of cell viability (2, 4, 6 and 24 h) was performed using the Alamar Blue assay in naïve IB3-1 *S1PR1^mut/mut^*, C38 *S1PR1^mut/mut^*, IB3-1 *S1PR1^wt/wt^* and C38 *S1PR1^wt/wt^* (**A**). Cells have been stimulated with exoproducts from *P. aeruginosa* AA2 strain supernatant (SN) (**B**). Data, from four independent experiments, are expressed as means ± standard errors of the means (SEM). The red dashed lines indicate the 100%, corresponding to the fluorescence of naïve cells at 20 min. ** *p* < 0.01, *** *p* < 0.001, **** *p* < 0.0001, according with the two-way ANOVA test with the Bonferroni correction for multiple comparisons.

Next, we investigated the role of the S1PR1 protein in both baseline conditions and in the responses induced by *P. aeruginosa* infection. Specifically, Clone 8 (IB3-1 S1PR1^mut/mut^) and clone 23 (C38 S1PR1^mut/mut^) and their isogenic S1PR1^wt/wt^ cell lines were exposed to the exoproducts of a clinical strain of *P. aeruginosa* known as AA2 [20]. Our aim was to assess both cell viability, cytotoxicity and cytokine secretion at baseline and following bacterial stimulation. As a control, we used stimulation with the bacterial culture medium, named tryptic soy broth (TSB). Cell viability and cytotoxicity was quantified by measuring metabolic activity with the Alamar Blue assay. First, metabolic activity was significantly lower in naïve IB3-1 *S1PR1^mut/mut^* cells compared to their isogenic *S1PR1^wt/wt^* counterpart (Figure 3A), consistent with increased baseline cytotoxicity in the CFTR-deficient background. A similar trend was observed in cells exposed to the TSB control (Supplementary Figure S1), indicating a role of S1PR1 in determining cell viability and cytotoxicity under basal conditions. When comparing the two genetic backgrounds, the effect of S1PR1 mutation was evident only in the CFTR-deficient IB3-1 cells, whereas no significant differences were observed between *S1PR1^mut/mut^* and *S1PR1^mut/mut^* C38 cells. This finding strenghtens the role of CFTR in determining the protective effects of S1PR1 against cellular cytotoxicity and in maintaining cell viability.

Stimulation with *P. aeruginosa* exoproducts resulted in a further decrease in cell viability in the IB3-1 *S1PR1^mut/mut^* cell line compared to naïve (Supplementary Figure S1). Under these conditions, the IB3-1 *S1PR1^mut/mut^* cell line exhibited a significantly increased difference compared to *S1PR1^wt/wt^* and other cell lines, including C38 *S1PR1^wt/wt^* and C38- *S1PR1^mut/mut^* (Figure 3B). No significant differences were observed in IB3-1 *S1PR1^wt/wt^* or in C38 cells, either *S1PR1^mut/mut^* or *S1PR1^wt/wt^*. These findings indicate that S1PR1 mutation increases baseline cytotoxicity in the CFTR-deficient genetic background, and that *P. aeruginosa* exoproducts further exacerbate this phenotype.

**Figure 4:**
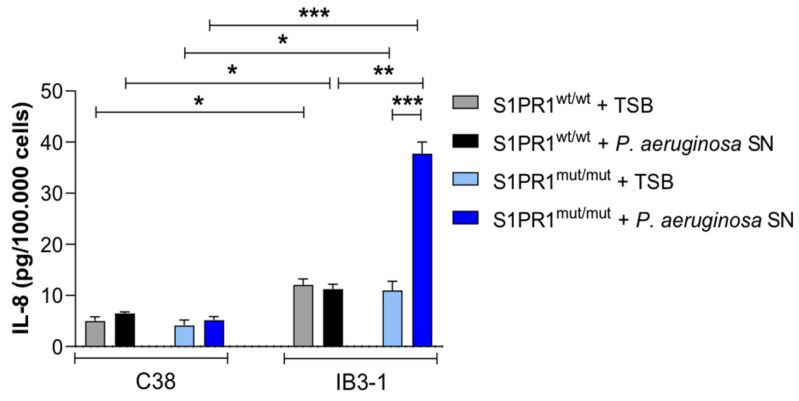
IL-8 release in IB3-1 and C38 cell lines, both *S1PR1^mut/mut^* and *S1PR1^wt/wt^*. IL-8 release was assessed by ELISA after stimulation with the exoproducts of the *P. aeruginosa* strain AA2 added to the cell culture medium. Data, from four independent experiments, are expressed as mean ± standard error of the mean (SEM). * *p* ≤ 0.05, ** *p* < 0.01, *** *p* < 0.001, according with the one-way ANOVA test with the Šidák correction for multiple comparisons.

Next, we evaluated the role of S1PR1 in modulating cytokine release, with a particular focus on IL-8, a cytokine responsible for recruiting neutrophils during lung infections. To do this, we stimulated the cell lines by adding the exoproducts of the *P. aeruginosa* strain AA2 into the cell culture medium. As a control, we subjected the same cell lines to a similar treatment, but added only the bacterial culture medium. Following a 24-h incubation period, we observed distinct IL-8 cytokine secretion profiles using the ELISA assay. In the CFTR-deficient background, S1PR1 mutation was associated with higher IL-8 levels upon exoproduct stimulation in the IB3-1 *S1PR1^mut/mut^* cell line, while C38 cells remained unaffected (Figure 4).

### 3.4. Protein Expression of S1PR1 in Lung Tissues of pwCF and Non-CF Individuals

To translate our findings to humans, we verified the presence of S1PR1 in lung tissues obtained from pwCF in comparison to non-CF individuals. Specifically, for this study, we selected 5 pwCF, all of whom were homozygous for the F508del mutation in *CFTR*, with one exception where the subject was heterozygous. The F508del is a processing mutation that removes a single amino acid from the CFTR protein, leading to mistrafficking and incorrect folding. The R1162X is a nonsense mutation containing an early stop signal that causes the production of the CFTR protein to stop prematurely. These individuals represented severe clinical conditions in both cases. They were compared with non-CF individuals (Table 2), all of whom had undergone transplantation and were in severely compromised health conditions for non-CF pathologies.

**Figure 5:**
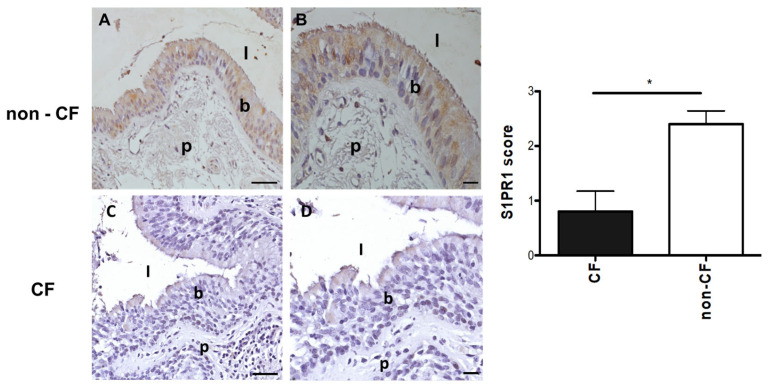
Staining for S1PR1 in human bronchial tissues. Immunohistochemical analysis of S1PR1 was conducted in human bronchial tissues obtained from non-CF individuals (*n* = 5) and pwCF (*n* = 5). Representative images show positive staining with the anti-S1PR1 antibody in non-CF airways, as seen in the individual BE67 ((**A**), 20×; (**B**), 40×), who has a wild-type CFTR. In contrast, the staining is negative in BE79, homozygous for F508del mutation ((**C**), 20×; (**D**), 40×). Data in the graph represent the score assigned to the presence of S1PR1 in the lung sections from CF (*n* = 5) and non-CF individuals (*n* = 5) and are expressed as means ± standard errors of the means (SEM). * *p* ≤ 0.05, two-tailed paired samples *t*-test. (**A**,**C**): scale bar: 50 um; (**B**,**D**): scale bar: 25 um. l: lumen; b: bronchial cells; p: parenchyma.

In the tissues, we evaluated the level and localization of the S1PR1 protein through immunohistochemistry. Our immunohistochemistry study successfully identified S1PR1 protein expression in the alveolar epithelium, lung vascular endothelium, and bronchial epithelial cells (Figure 5). However, we observed that signals for S1PR1 protein were either absent or significantly reduced in the lungs of CF individuals in comparison to non-CF ones.

## 4. Discussion

CF is characterized by considerable phenotypic variability, even among individuals carrying the same CFTR mutations, and numerous studies have demonstrated that this heterogeneity is partly attributable to modifier genes. Genes such as *TGFB1* (involved in inflammation and fibrosis regulation), *IL8* (cytokine signaling), *EDNRA* (vascular tone and remodeling), *EHF* and *APIP* (oxidative stress response), and *MUC5B* and *SLC9A3* (airway mucus and ion transport) have been associated with variations in lung function, infection susceptibility, and inflammation in pwCF [6,25,26]. These genetic factors highlight the role of the host background in modulating epithelial stress responses and inflammatory signaling. Within this framework, S1PR1 emerges as a novel candidate modifier gene potentially influencing the pulmonary phenotype in CF. S1PR1 mediates several physiological processes and plays a central role in immune and vascular regulation. S1PR1 is ubiquitous and is expressed in nearly every cell line [27], but it is especially abundant and extensively studied in immune cells and endothelial cells, while its role in epithelial cells remains unclear. S1PR1’s broad distribution underlies its pleiotropic effects across various organs [16,28,29]. At the peripheral level, immune and vascular cells carry out multiple functions such as actin remodeling, chemotaxis, lymphocyte egress, vascular integrity, organogenesis, including angiogenesis, cell growth and proliferation, and immune response [30]. However, the role of S1PR1 in the context of infection and respiratory diseases, especially CF, has not been fully elucidated.

This study aimed to specifically investigate the role of S1PR1 in the context of CFTR deficiency and bacterial challenge. Thanks to gene editing based on CRISPR/Cas9 technology and the use of lentiviral vectors, we engineered the two human bronchial epithelial IB3-1 and C38 cell lines and successfully introduced mutations in S1PR1. The *S1PR1* gene consists of two exons, with only exon 2 being protein-coding, thus making it the selected target for CRISPR/Cas9 guides. As a result, we generated a CFTR-mutant cell line with mutations in *S1PR1* (IB3-1 *S1PR1^mut/mut^*) and a wild-type CFTR cell line also carrying S1PR1 mutations (C38 *S1PR1^mut/mut^*). This pairwise setup enabled us to evaluate the role of S1PR1 in the presence or absence of CFTR dysfunction. Matched controls were also generated via transduction with an empty lentiviral vector to obtain *S1PR1^wt/wt^* cells.

The immunofluorescence staining indicates that S1PR1 in C38 cells (wild-type CFTR) is broadly distributed throughout the cell. However, after mutation, S1PR1 is predominantly localized in a perinuclear position, suggesting the presence of a non-functional protein in compartments such as the Golgi apparatus or endoplasmic reticulum. In the case of defective S1PR1, it would likely undergo degradation and be internalized by lysosomes. Supporting this, Chavez et al. have observed that phosphorylation on tyrosine 143 of S1PR1 in endothelial cells leads to its internalization and inhibition of its binding with S1P [31]. Mutations in S1PR1 at tyrosine 143 internalize the protein, sequestering it in the cytoplasm, with results highly similar to the findings obtained in this work. Notably, in IB3-1 cells (CFTR-mutant), the localization of even wt S1PR1 protein shows partial perinuclear accumulation, and the mutation further intensifies mislocalization. Sequence analysis revealed that mutations of *S1PR1* in IB3-1 produced several peptides of varying lengths, which primarily relocate and accumulate in the perinuclear cytoplasmic region. These results suggest that *S1PR1* mutations disrupt protein processing and trafficking, leading to additional cellular stress and pathology beyond the effects of CFTR mutations. Our data, using the Alamar Blue assay, showed that *S1PR1* mutation in the *CFTR*-deficient genetic background (IB3-1) reduces metabolic activity under basal conditions, thereby affecting cell viability and increasing cytotoxicity. These effects are further exacerbated upon exposure to *P. aeruginosa* exoproducts, highlighting the impact on epithelial resilience to infection.

It is known in the literature that the *F508del* mutation retains the CFTR protein in the endoplasmic reticulum in a glycosylated and immature form, which is rapidly degraded intracellularly. This prevents its localization in the plasma membrane [32,33]. These phenomena lead to intracellular stress, affecting the maturation of proteins and likely S1PR1 as well. Our study utilizes the IB3-1 human bronchial epithelial cell line derived from a person with CF, specifically deficient for *CFTR* (*F508del/W1282X*), along with its corrected counterpart, C38. Theoretically, both cell lines retain the mutated CFTR protein in the endoplasmic reticulum, leading to similar endoplasmatic stress. However, the presence of *wt/wt CFTR* in C38 cells, unlike in IB3-1 cells, may rescue other cellular stress factors such as elevated oxidative stress [34] and abnormal lipid metabolism [35]. Indeed, it is well known that the inactivity of the channel, as well as bacterial infections, is also responsible for producing a redox imbalance in epithelial cells and alterations in the lipid composition of the plasma membrane. Notably, the sphingolipid-richlipid rafts are organizing platforms where specific proteins involved in the signaling mediated across the plasma membrane are recruited, including S1PR1 [36]. Consequently, it is plausible that CF cells may experience impaired S1PR1 stability and activity due to these intricate alterations in lipid composition and cellular processes. Future studies could provide a more detailed characterization of the mechanisms through which S1PR1 impacts the pathophysiology of the CF epithelium and host defenses during bacterial infections.

Previous studies have consistently demonstrated that the dysfunction of the S1PR1 receptor signaling system leads to heightened inflammation primarily as a consequence of increased endothelial barrier permeability. Recently, Hao et al. [17] reported that S1PR1 is downregulated in the lungs of people with idiopathic pulmonary fibrosis and the endothelial-conditional S1PR1 knockout mouse model exhibited inflammation and fibrosis. The lack of S1PR1 in the vascular endothelium results in a ruptured endothelial barrier, increased inflammation and collagen deposition. Our study has further established a connection between S1PR1 and CFTR: in CFTR-mutant bronchial cells with mutated S1PR1, exposure to *P. aeruginosa* exoproducts triggered a pronounced IL-8 response, suggesting that S1PR1 influences the host’s inflammatory reactivity to pathogens in a CF-dependent manner. Undoubtedly, the presence or absence of S1PR1 is a variable implicated in inflammation and its associated events, such as the secretion of inflammatory cytokines like IL-8. Simultaneously, the inflammatory pathway of S1PR1 seems to be intricately linked to the mutation in CFTR.

Finally, immunohistochemistry on tissues from non-CF individuals revealed the expression of the S1PR1 protein in bronchial epithelial cells, alveolar pulmonary epithelium, and vascular endothelium, whereas lungs from pwCF exhibited a marked reduction or almost complete absence of signal. This observation is consistent with the fact that the CF tissues analyzed were obtained from explanted lungs of pwCF with advanced disease and severely impaired respiratory function. In such cases, the presence of mutations or regulatory alterations in S1PR1 may have led to markedly reduced protein expression, which in turn could have been associated with more severe clinical manifestations. It is plausible that, in pwCF with milder disease, S1PR1 expression might be comparatively higher, reflecting a less compromised regulatory balance. Future studies including tissues from pwCF with different degrees of disease severity will be instrumental in clarifying the relationship between S1PR1 expression, genetic variation, and CF progression.

Overall, these findings support the concept that S1PR1 acts as a modifier influencing the severity of CF lung disease and shaping the epithelial response to bacterial infection. A deeper understanding of this pathway could provide new perspectives for therapeutic modulation of inflammation and host–pathogen interactions in CF.

## Figures and Tables

**Table 1:** The 20-nucleotide protospacer sequences of the guides (sgRNA) designed to target the second exon of the human *S1PR1* gene.

sgRNA ID	Target Sequence
W3	CGTAGTCAGAGACCGAGCTG
W6	GATGGCGAGGAGACTGAACA
W9	ATATAGTGCTTGTGGTAGAG

**Table 2:** List of pwCF and non-CF individuals, including CFTR genotype, analyzed in the study.

Individual	Pathology	CFTR Mutations
BE 55	CF	F508del/F508del
BE 60	CF	F508del/R1162X
BE 68	CF	F508del/F508del
BE 73	CF	F508del/F508del
BE 79	CF	F508del/F508del
BE 49	non-CF	-
BE 50	non-CF	-
BE 65	non-CF	-
BE 66	non-CF	-
BE 67	non-CF	-

## Data Availability

Sequencing data are publicly available at ENA under the project number PRJEB76287 samples ERS20182475 (C38 clone 23) and ERS20182474 (IB3-1 clone 8).

## Supplementary Materials

The following supporting information can be downloaded at: https://www.mdpi.com/article/10.3390/pathogens14111146/s1, Table S1: PCR primers used for the generation of amplicons for amplicon sequencing; Supplementary Alignments S1; Figure S1: Cell viability after *P. aeruginosa* stimulation.

## Abbreviations

The following abbreviations are used in this manuscript:

BSA	bovine serum albumin
CF	cystic fibrosis
CFTR	cystic fibrosis transmembrane conductance regulator
DAPI	4,6-Diamidino-2-phenylindole dihydrochloride
IL-8	interleukin-8
MOI	multiplicity of infection
OD	optical density
O/N	overnight
PBS	phosphate-buffered saline
PFA	paraformaldehyde
pwCF	people with CF
S1PR1	sphingosine 1-phosphate receptor 1
SEM	standard errors of the means
sgRNA	single guide RNA
SN	supernatant
TSB	tryptic soy broth
