# Supplementary material for: Role of S1PR1 in Modulating Airway Epithelial Responses to Pseudomonas aeruginosa in Cystic Fibrosis
Source: Pathogens. 2025 Nov 12;14(11):1146. doi: 10.3390/pathogens14111146 (PMC12655217; doi:10.3390/pathogens14111146)
Supplement: Supplementary file 1 [file pathogens-14-01146-s001.zip › pathogens-3925176-supplementary.pdf]

Supplementary Table S1 PCR primers used for the generation of amplicons for amplicon sequencing. Tm: melting temperature. Primers were designed using the PrimerQuest Tool available on the Integrated DNA Technologies (IDT) platform using default parameters.

	Sequence (5′-3′)	Start	Stop	Length	Tm	GC%
Forward	TCTAGCGTTCGTCTGGAGTAG	199	220	21	62	52.4
Reverse	GGAACTAACTCTACCCACCAAC	1565	1587	22	62	50

Supplementary Alignments S1. Nucleotide and protein alignments of C38 clone alleles (**S1A** and **S1B**) and IB3-1 clone alleles (**S1C** and **S1D**) to wild-type S1PR1. Matches with the wild-type sequence are indicated in green, deletions are indicated in red, protein portions recognized by the antibody EDG-1/S1P1/S1PR1 (A-6) | SCBT - Santa Cruz Biotechnology are highlighted in yellow. The sequences of all possible peptides generated by each allele and the lost transmembrane domains (annotations available at: https://www.uniprot.org/uniprotkb/P21453/entry) are reported at the end of the alignments.

<u>S1A C38 allele 1</u>

```
7236
atg ggg ccc acc agc gtc ccg ctg gtc aag gcc cac cgc agc tcg gtc tct gac tac gtc
 M   G   P   T   S   V   P   L   V   K   A   H   R   S   S   V   S   D   Y   V
aac tat gat atc atc gtc cgg cat tac aac tac acg gga aag ctg aat atc agc gcg gac
 N   Y   D   I   I   V   R   H   Y   N   Y   T   G   K   L   N   I   S   A   D
aag gag aac agc att aaa ctg acc tcg gtg gtg ttc att ctc atc tgc tgc ttt atc atc
 K   E   N   S   I   K   L   T   S   V   V   F   I   L   I   C   C   F   I   I
ctg gag aac atc ttt gtc ttg ctg acc att tgg aaa acc aag aaa ttc cac cga ccc atg
 L   E   N   I   F   V   L   L   T   I   W   K   T   K   K   F   H   R   P   M
tac tat ttt att ggc aat ctg gcc ctc tca gac ctg ttg gca gga gta gcc tac aca gct
 Y   Y   F   I   G   N   L   A   L   S   D   L   L   A   G   V   A   Y   T   A
aac ctg ctc ttg tct ggg gcc acc acc tac aag ctc act ccc gcc cag tgg ttt ctg cgg
 N   L   L   L   S   G   A   T   T   Y   K   L   T   P   A   Q   W   F   L   R
gaa ggg agt atg ttt gtg gcc ctg tca gcc tcc gtg ttc agt ctc ctc gcc atc gcc att
 E   G   S   M   F   V   A   L   S   A   S   V   F   S   L   L   A   I   A   I
gag cgc tat atc aca atg ctg aaa atg aaa ctc cac aac ggg agc aat aac ttc cgc ctc
 E   R   Y   I   T   M   L   K   M   K   L   H   N   G   S   N   N   F   R   L
ttc ctg cta atc agc gcc tgc tgg gtc atc tcc ctc atc ctg ggt ggc ctg cct atc atg
 F   L   L   I   S   A   C   W   V   I   S   L   I   L   G   G   L   P   I   M
ggc tgg aac tgc atc agt gcg ctg tcc agc tgc tcc acc gtg ctg ccg ctc tac cac aag
 G   W   N   C   I   S   A   L   S   S   C   S   T   V   L   P   L   Y   H   K
cac tat atc ctc ttc tgc acc acg gtc ttc act ctg ctt ctg ctc tcc atc gtc att ctg
 H   Y   I   L   F   C   T   T   V   F   T   L   L   L   L   S   I   V   I   L
tac tgc aga atc tac tcc ttg gtc agg act cgg agc cgc cgc ctg acg ttc cgc aag aac
 Y   C   R   I   Y   S   L   V   R   T   R   S   R   R   L   T   F   R   K   N
att tcc aag gcc agc cgc agc tct gag aag tcg ctg gcg ctg ctc aag acc gta att atc
 I   S   K   A   S   R   S   S   E   K   S   L   A   L   L   K   T   V   I   I
gtc ctg agc gtc ttc atc gcc tgc tgg gca ccg ctc ttc atc ctg ctc ctg ctg gat gtg
 V   L   S   V   F   I   A   C   W   A   P   L   F   I   L   L   L   L   D   V
ggc tgc aag gtg aag acc tgt gac atc ctc ttc aga gcg gag tac ttc ctg gtg tta gct
 G   C   K   V   K   T   C   D   I   L   F   R   A   E   Y   F   L   V   L   A
gtg ctc aac tcc ggc acc aac ccc atc att tac act ctg acc aac aag gag atg cgt cgg
 V   L   N   S   G   T   N   P   I   I   Y   T   L   T   N   K   E   M   R   R
gcc ttc atc cgg atc atg tcc tgc tgc aag tgc ccg agc gga gac tct gct ggc aaa ttc
 A   F   I   R   I   M   S   C   C   K   C   P   S   G   D   S   A   G   K   F
aag cga ccc atc atc gcc ggc atg gaa ttc agc cgc agc aaa tcg gac aat tcc tcc cac
 K   R   P   I   I   A   G   M   E   F   S   R   S   K   S   D   N   S   S   H
ccc cag aaa gac gaa ggg gac aac cca gag acc att atg tct tct gga aac gtc aac tct
 P   Q   K   D   E   G   D   N   P   E   T   I   M   S   S   G   N   V   N   S
tct tcc tag 8385
 S   S   -
```

Peptides sequences:

<u>5'3' Frame 1 (lost transmembrane domains: T1, T2, T3)</u>

MGPTSVPLVKAHLILGGLPIMGWNCISALSSCSTVLPLYHKHYILFCTTVFTLLLLSIVILYCRIYSLVRTRSRRLTFRKNISKASRSSEKSLALLKTVIIVLSVFIACWAPLFILLLLDVGCKVKTCDILFRAEYFLVLAVLNSGTNPIIYTLTNKEMRRAFIRIMSCCKCPSGDSAGKFKRPIIAGMEFSRSKSDNSSHPQKDEGDNPETIMSSGNVNSSS

<u>5'3' Frame 2 (lost transmembrane domains: T1, T2, T3, T4, T5, T6, T7)</u>

MWAAR-

<u>3'5' Frame 2 (lost transmembrane domains: T1, T2, T3, T4, T5, T6, T7)</u>

MVSGLSPSSFWGWEELSDLLRLNSMPAMMGRLNLPAESPLGHLQQDMIRMKARRISLLVRV-MMGLVPELSTANTRKYSALKRMSQVFTLQPTSSRSRMKSGAQQAMKTLRTIITVLSSASDFSELRLALEMFLRNVRRRLRVLTKE-

MTMESRSRVKTVVQKRI-
MQFQPMIGRPPRMRWALTSGTLVGP

S1B C38 allele 2

```
7236
atg ggg ccc acc agc gtc ccg ctg gtc aag gcc cac cgc agc tcg gtc tct gac tac gtc
 M   G   P   T   S   V   P   L   V   K   A   H   R   S   S   V   S   D   Y   V
aac tat gat atc atc gtc cgg cat tac aac tac acg gga aag ctg aat atc agc gcg gac
 N   Y   D   I   I   V   R   H   Y   N   Y   T   G   K   L   N   I   S   A   D
aag gag aac agc att aaa ctg acc tcg gtg gtg ttc att ctc atc tgc tgc ttt atc atc
 K   E   N   S   I   K   L   T   S   V   V   F   I   L   I   C   C   F   I   I
ctg gag aac atc ttt gtc ttg ctg acc att tgg aaa acc aag aaa ttc cac cga ccc atg
 L   E   N   I   F   V   L   L   T   I   W   K   T   K   K   F   H   R   P   M
tac tat ttt att ggc aat ctg gcc ctc tca gac ctg ttg gca gga gta gcc tac aca gct
 Y   Y   F   I   G   N   L   A   L   S   D   L   L   A   G   V   A   Y   T   A
aac ctg ctc ttg tct ggg gcc acc acc tac aag ctc act ccc gcc cag tgg ttt ctg cgg
 N   L   L   L   S   G   A   T   T   Y   K   L   T   P   A   Q   W   F   L   R
gaa ggg agt atg ttt gtg gcc ctg tca gcc tcc gtg ttc agt ctc ctc gcc atc gcc att
 E   G   S   M   F   V   A   L   S   A   S   V   F   S   L   L   A   I   A   I
gag cgc tat atc aca atg ctg aaa atg aaa ctc cac aac ggg agc aat aac ttc cgc ctc
 E   R   Y   I   T   M   L   K   M   K   L   H   N   G   S   N   N   F   R   L
ttc ctg cta atc agc gcc tgc tgg gtc atc tcc ctc atc ctg ggt ggc ctg cct atc atg
 F   L   L   I   S   A   C   W   V   I   S   L   I   L   G   G   L   P   I   M
ggc tgg aac tgc atc agt gcg ctg tcc agc tgc tcc acc gtg ctg ccg ctc tac cac aag
 G   W   N   C   I   S   A   L   S   S   C   S   T   V   L   P   L   Y   H   K
cac tat atc ctc ttc tgc acc acg gtc ttc act ctg ctt ctg ctc tcc atc gtc att ctg
 H   Y   I   L   F   C   T   T   V   F   T   L   L   L   L   S   I   V   I   L
tac tgc aga atc tac tcc ttg gtc agg act cgg agc cgc cgc ctg acg ttc cgc aag aac
 Y   C   R   I   Y   S   L   V   R   T   R   S   R   R   L   T   F   R   K   N
att tcc aag gcc agc cgc agc tct gag aag tcg ctg gcg ctg ctc aag acc gta att atc
 I   S   K   A   S   R   S   S   E   K   S   L   A   L   L   K   T   V   I   I
gtc ctg agc gtc ttc atc gcc tgc tgg gca ccg ctc ttc atc ctg ctc ctg ctg gat gtg
 V   L   S   V   F   I   A   C   W   A   P   L   F   I   L   L   L   L   D   V
ggc tgc aag gtg aag acc tgt gac atc ctc ttc aga gcg gag tac ttc ctg gtg tta gct
 G   C   K   V   K   T   C   D   I   L   F   R   A   E   Y   F   L   V   L   A
gtg ctc aac tcc ggc acc aac ccc atc att tac act ctg acc aac aag gag atg cgt cgg
 V   L   N   S   G   T   N   P   I   I   Y   T   L   T   N   K   E   M   R   R
gcc ttc atc cgg atc atg tcc tgc tgc aag tgc ccg agc gga gac tct gct ggc aaa ttc
 A   F   I   R   I   M   S   C   C   K   C   P   S   G   D   S   A   G   K   F
aag cga ccc atc atc gcc ggc atg gaa ttc agc cgc agc aaa tcg gac aat tcc tcc cac
 K   R   P   I   I   A   G   M   E   F   S   R   S   K   S   D   N   S   S   H
ccc cag aaa gac gaa ggg gac aac cca gag acc att atg tct tct gga aac gtc aac tct
 P   Q   K   D   E   G   D   N   P   E   T   I   M   S   S   G   N   V   N   S
tct tcc tag 8385
 S   S   -
```

Peptides sequences:

5'3' Frame 1 (lost transmembrane domains: T1, T2, T3, T4, T5, T6, T7)

MGPTSVPLVKAHRSTTSTISSSAPRSSLCFCSPSSFCTAESTPWSGLGAAA-

5'3' Frame 3 (lost transmembrane domains: T1, T2, T3, T4, T5, T6, T7)

MRRAFIRIMSCCKCPSGDSAGKFKRPIIAGMEFSRSKSDNSSHPQKDEGDNPETIMSSGNVNSSS-

3'5' Frame 2 (lost transmembrane domains: T1, T2, T3, T4, T5, T6, T7)

MVSGLSPSSFWGWEELSDLLRLNSMPAMMGRLNLPAESPLGHLQQDMIRMKARRISLLVRV-
MMGLVPELSTANTRKYSALKRMSQVFTLQPTSSRSRMKSGAQQAMKTLRTIITVLSSASDFSELRLALEMFLRNVRRRLR
VLTKE-
MTMESRSRVKTVVQKRI-

S1C IB3-1 allele 1

```
7236
atg ggg ccc acc agc gtc ccg ctg gtc aag gcc cac cgc agc tcg gtc tct gac tac gtc
 M   G   P   T   S   V   P   L   V   K   A   H   R   S   S   V   S   D   Y   V
```

```
aac tat gat atc atc gtc cgg cat tac aac tac acg gga aag ctg aat atc agc gcg gac
 N   Y   D   I   I   V   R   H   Y   N   Y   T   G   K   L   N   I   S   A   D
aag gag aac agc att aaa ctg acc tcg gtg gtg ttc att ctc atc tgc tgc ttt atc atc
 K   E   N   S   I   K   L   T   S   V   V   F   I   L   I   C   C   F   I   I
ctg gag aac atc ttt gtc ttg ctg acc att tgg aaa acc aag aaa ttc cac cga ccc atg
 L   E   N   I   F   V   L   L   T   I   W   K   T   K   K   F   H   R   P   M
tac tat ttt att ggc aat ctg gcc ctc tca gac ctg ttg gca gga gta gcc tac aca gct
 Y   Y   F   I   G   N   L   A   L   S   D   L   L   A   G   V   A   Y   T   A
aac ctg ctc ttg tct ggg gcc acc acc tac aag ctc act ccc gcc cag tgg ttt ctg cgg
 N   L   L   L   S   G   A   T   T   Y   K   L   T   P   A   Q   W   F   L   R
gaa ggg agt atg ttt gtg gcc ctg tca gcc tcc gtg ttc agt ctc ctc gcc atc gcc att
 E   G   S   M   F   V   A   L   S   A   S   V   F   S   L   L   A   I   A   I
gag cgc tat atc aca atg ctg aaa atg aaa ctc cac aac ggg agc aat aac ttc cgc ctc
 E   R   Y   I   T   M   L   K   M   K   L   H   N   G   S   N   N   F   R   L
ttc ctg cta atc agc gcc tgc tgg gtc atc tcc ctc atc ctg ggt ggc ctg cct atc atg
 F   L   L   I   S   A   C   W   V   I   S   L   I   L   G   G   L   P   I   M
ggc tgg aac tgc atc agt gcg ctg tcc agc tgc tcc acc gtg ctg ccg ctc tac cac aag
 G   W   N   C   I   S   A   L   S   S   C   S   T   V   L   P   L   Y   H   K
cac tat atc ctc ttc tgc acc acg gtc ttc act ctg ctt ctg ctc tcc atc gtc att ctg
 H   Y   I   L   F   C   T   T   V   F   T   L   L   L   L   S   I   V   I   L
tac tgc aga atc tac tcc ttg gtc agg act cgg agc cgc cgc ctg acg ttc cgc aag aac
 Y   C   R   I   Y   S   L   V   R   T   R   S   R   R   L   T   F   R   K   N
att tcc aag gcc agc cgc agc tct gag aag tcg ctg gcg ctg ctc aag acc gta att atc
 I   S   K   A   S   R   S   S   E   K   S   L   A   L   L   K   T   V   I   I
gtc ctg agc gtc ttc atc gcc tgc tgg gca ccg ctc ttc atc ctg ctc ctg ctg gat gtg
 V   L   S   V   F   I   A   C   W   A   P   L   F   I   L   L   L   L   D   V
ggc tgc aag gtg aag acc tgt gac atc ctc ttc aga gcg gag tac ttc ctg gtg tta gct
 G   C   K   V   K   T   C   D   I   L   F   R   A   E   Y   F   L   V   L   A
gtg ctc aac tcc ggc acc aac ccc atc att tac act ctg acc aac aag gag atg cgt cgg
 V   L   N   S   G   T   N   P   I   I   Y   T   L   T   N   K   E   M   R   R
gcc ttc atc cgg atc atg tcc tgc tgc aag tgc ccg agc gga gac tct gct ggc aaa ttc
 A   F   I   R   I   M   S   C   C   K   C   P   S   G   D   S   A   G   K   F
aag cga ccc atc atc gcc ggc atg gaa ttc agc cgc agc aaa tcg gac aat tcc tcc cac
 K   R   P   I   I   A   G   M   E   F   S   R   S   K   S   D   N   S   S   H
ccc cag aaa gac gaa ggg gac aac cca gag acc att atg tct tct gga aac gtc aac tct
 P   Q   K   D   E   G   D   N   P   E   T   I   M   S   S   G   N   V   N   S
tct tcc tag 8385
 S   S   -
```

Peptides sequences:

5'3' Frame 1 (lost transmembrane domains: T1, T2, T3, T4, T5, T6, T7)

MGPTSVPLVKAHRSTTSTISSSAPRSSLCFCSPSSFCTAESTPWSGLGAAA-

5'3' Frame 3 (lost transmembrane domains: T1, T2, T3, T4, T5, T6, T7)

MRRAFIRIMSCCKCPSGDSAGKFKRPIIAGMEFSRSKSDNSSHPQKDEGDNPETIMSSGNVNSSS-

3'5' Frame 2 (lost transmembrane domains: T1, T2, T3, T4, T5, T6, T7)

MVSGLSPSSFWGWEELSDLLRLNSMPAMMGRLNLPAESPLGHLQQDMIRMKARRISLLVRV-

MMGLVPELSTANTRKYSALKRMSQVFTLQPTSSRSRMKSGAQQAMKTLRTIITVLSSASDFSELRLALEMFLRNVRRRLRVLTKE-

MTMESRSRVKTVVQKRI

S1D IB3-1 allele 2

```
7236
atg ggg ccc acc agc gtc ccg ctg gtc aag gcc cac cgc agc tcg gtc tct gac tac gtc
 M   G   P   T   S   V   P   L   V   K   A   H   R   S   S   V   S   D   Y   V
aac tat gat atc atc gtc cgg cat tac aac tac acg gga aag ctg aat atc agc gcg gac
 N   Y   D   I   I   V   R   H   Y   N   Y   T   G   K   L   N   I   S   A   D
aag gag aac agc att aaa ctg acc tcg gtg gtg ttc att ctc atc tgc tgc ttt atc atc
 K   E   N   S   I   K   L   T   S   V   V   F   I   L   I   C   C   F   I   I
ctg gag aac atc ttt gtc ttg ctg acc att tgg aaa acc aag aaa ttc cac cga ccc atg
 L   E   N   I   F   V   L   L   T   I   W   K   T   K   K   F   H   R   P   M
tac tat ttt att ggc aat ctg gcc ctc tca gac ctg ttg gca gga gta gcc tac aca gct
 Y   Y   F   I   G   N   L   A   L   S   D   L   L   A   G   V   A   Y   T   A
```

```
aac ctg ctc ttg tct ggg gcc acc acc tac aag ctc act ccc gcc cag tgg ttt ctg cgg
 N   L   L   L   S   G   A   T   T   Y   K   L   T   P   A   Q   W   F   L   R
gaa ggg agt atg ttt gtg gcc ctg tca gcc tcc gtg ttc agt ctc ctc gcc atc gcc att
 E   G   S   M   F   V   A   L   S   A   S   V   F   S   L   L   A   I   A   I
gag cgc tat atc aca atg ctg aaa atg aaa ctc cac aac ggg agc aat aac ttc cgc ctc
 E   R   Y   I   T   M   L   K   M   K   L   H   N   G   S   N   N   F   R   L
ttc ctg cta atc agc gcc tgc tgg gtc atc tcc ctc atc ctg ggt ggc ctg cct atc atg
 F   L   L   I   S   A   C   W   V   I   S   L   I   L   G   G   L   P   I   M
ggc tgg aac tgc atc agt gcg ctg tcc agc tgc tcc acc gtg ctg ccg ctc tac cac aag
 G   W   N   C   I   S   A   L   S   S   C   S   T   V   L   P   L   Y   H   K
cac tat atc ctc ttc tgc acc acg gtc ttc act ctg ctt ctg ctc tcc atc gtc att ctg
 H   Y   I   L   F   C   T   T   V   F   T   L   L   L   L   S   I   V   I   L
tac tgc aga atc tac tcc ttg gtc agg act cgg agc cgc cgc ctg acg ttc cgc aag aac
 Y   C   R   I   Y   S   L   V   R   T   R   S   R   R   L   T   F   R   K   N
att tcc aag gcc agc cgc agc tct gag aag tcg ctg gcg ctg ctc aag acc gta att atc
 I   S   K   A   S   R   S   S   E   K   S   L   A   L   L   K   T   V   I   I
gtc ctg agc gtc ttc atc gcc tgc tgg gca ccg ctc ttc atc ctg ctc ctg ctg gat gtg
 V   L   S   V   F   I   A   C   W   A   P   L   F   I   L   L   L   L   D   V
ggc tgc aag gtg aag acc tgt gac atc ctc ttc aga gcg gag tac ttc ctg gtg tta gct
 G   C   K   V   K   T   C   D   I   L   F   R   A   E   Y   F   L   V   L   A
gtg ctc aac tcc ggc acc aac ccc atc att tac act ctg acc aac aag gag atg cgt cgg
 V   L   N   S   G   T   N   P   I   I   Y   T   L   T   N   K   E   M   R   R
gcc ttc atc cgg atc atg tcc tgc tgc aag tgc ccg agc gga gac tct gct ggc aaa ttc
 A   F   I   R   I   M   S   C   C   K   C   P   S   G   D   S   A   G   K   F
aag cga ccc atc atc gcc ggc atg gaa ttc agc cgc agc aaa tcg gac aat tcc tcc cac
 K   R   P   I   I   A   G   M   E   F   S   R   S   K   S   D   N   S   S   H
ccc cag aaa gac gaa ggg gac aac cca gag acc att atg tct tct gga aac gtc aac tct
 P   Q   K   D   E   G   D   N   P   E   T   I   M   S   S   G   N   V   N   S
tct tcc tag 8385
 S   S   -
```

Peptides sequences:

5'3' Frame 1 (lost transmembrane domains: T1, T2, T3, T4, T5, T6, T7)

MGPTSVPLVKAHRSQL-

MWAAR-

5'3' Frame 3

MISSSGITTTRES-

MRRAFIRIMSCCKCPSGDSAGKFKRPIIAGMEFSRSKSDNSSHPQKDEGDNPETIMSSGNVNSSS-

3'5' Frame 2 (lost transmembrane domains: T1, T2, T3, T4, T5, T6, T7)

MVSGLSPSSFWGWEELSDLLRLNSMPAMMGRLNLPAESPLGHLQQDMIRMKARRISLLVRV-

MMGLVPELSTANTRKYSALKRMSQVFTLQPTSSRSRMKSGAQQAMKTLRTIITVLSSASDFSELRLALEMFLRNVRRRLR
VLTKE-

MTMESRSRVKTVVQKRI-

MPDDDIIVDCGGP-

3'5' Frame 3 (lost transmembrane domains: T1, T2, T3, T4, T5, T6, T7)

MLFSLSALIFSFPV-

MIS-

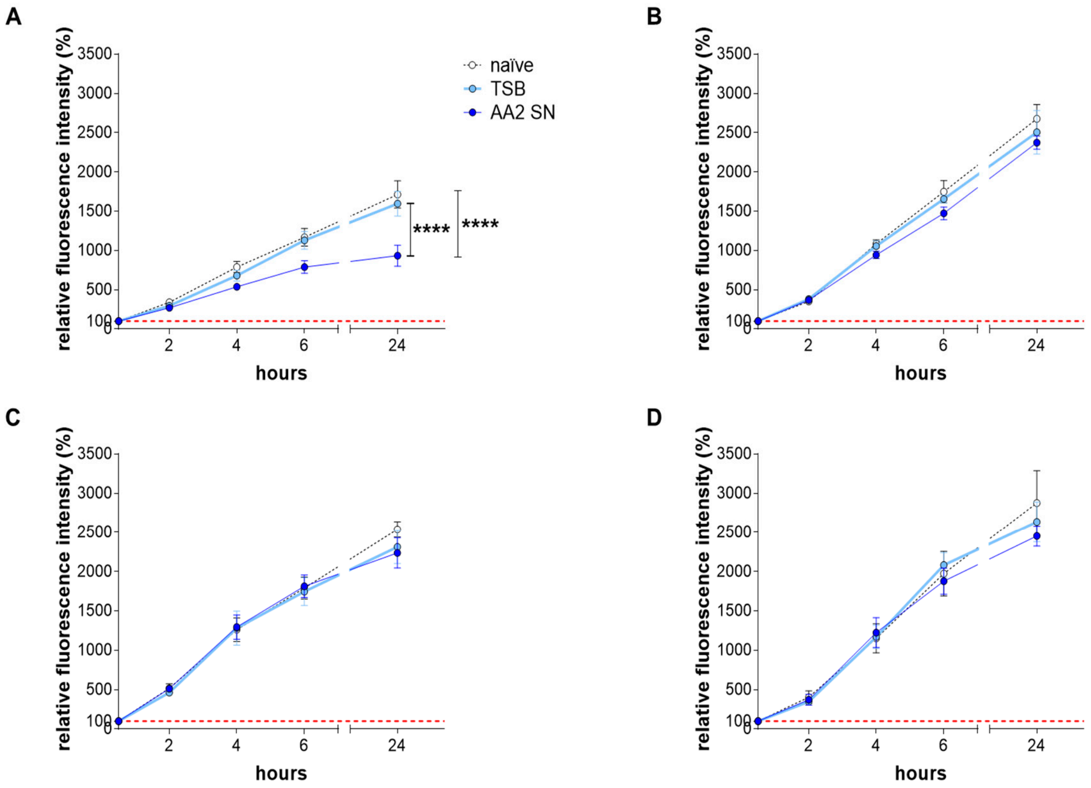

Supplementary Figure S1: Cell viability after *P. aeruginosa* stimulation. Cell viability was assessed by Alamar assay in the following cell lines: IB3-1 S1PR1$^{mut/mut}$ (A), C38 S1PR1$^{mut/mut}$ (B), IB3-1 S1PR1$^{wt/wt}$ and C38 S1PR1$^{wt/wt}$. A time-course analysis was conducted after stimulation with bacterial culture medium (TSB) and *P. aeruginosa* AA2 strain exoproducts released in TSB for 2, 4, 6 and 24 hours. Data, from four independent experiments, are expressed as means ± standard errors of the means (SEM). ****P<0.0001, two-way ANOVA test with the Bonferroni correction for multiple comparisons.
